# Supplementary material for: Paired and solitary ionocytes in the zebrafish olfactory epithelium
Source: Chem Senses. 2025 Aug 13;50:bjaf031. doi: 10.1093/chemse/bjaf031 (PMC12416361; doi:10.1093/chemse/bjaf031)
Supplement: bjaf031_suppl_Supplementary_Figures_S1-S4_Tables_S2 [file bjaf031_suppl_supplementary_figures_s1-s4_tables_s2.pdf]

Supplementary Material

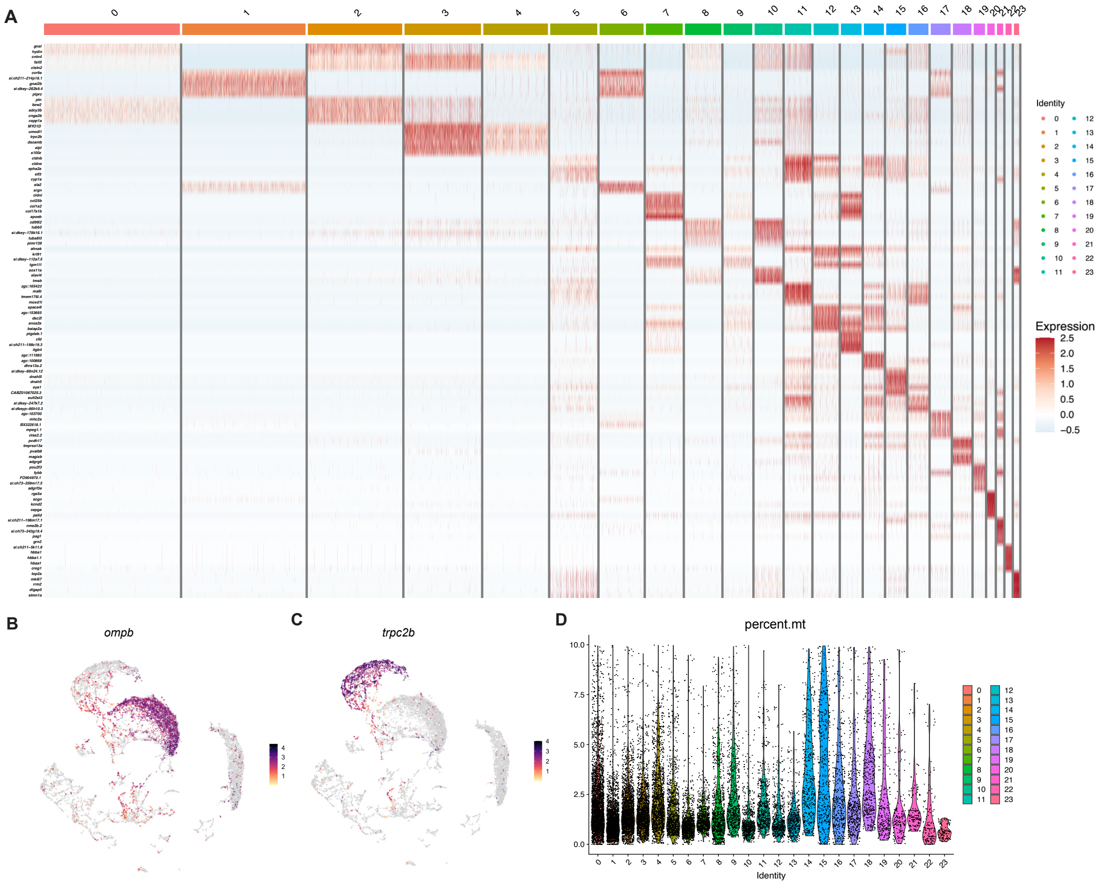

Figure S1, related to Figure 1

(A) Heatmap showing top 5 highest expressing genes based on LogFC on the clusters obtained in the dataset from dissected adult zebrafish olfactory organs. Based on these genes and markers given previously (Kraus et al., 2022), the clusters can be classified as: ciliated neurons: clusters 0, 2; microvillous neurons: clusters 3, 4; neuronal precursors: clusters 8, 10, 23; early progenitors: clusters 14, 15; sustentacular cells: clusters 7, 9, 12, 13, 20; immune cells: clusters 1, 6, 11, 17, 21. **(B)** Feature plots of *trpc2b* (microvillous OSN marker) and **(C)** *ompb* (ciliated

OSN marker). **(D)** Violin plot showing the percentage of mitochondrial genes in the dataset.

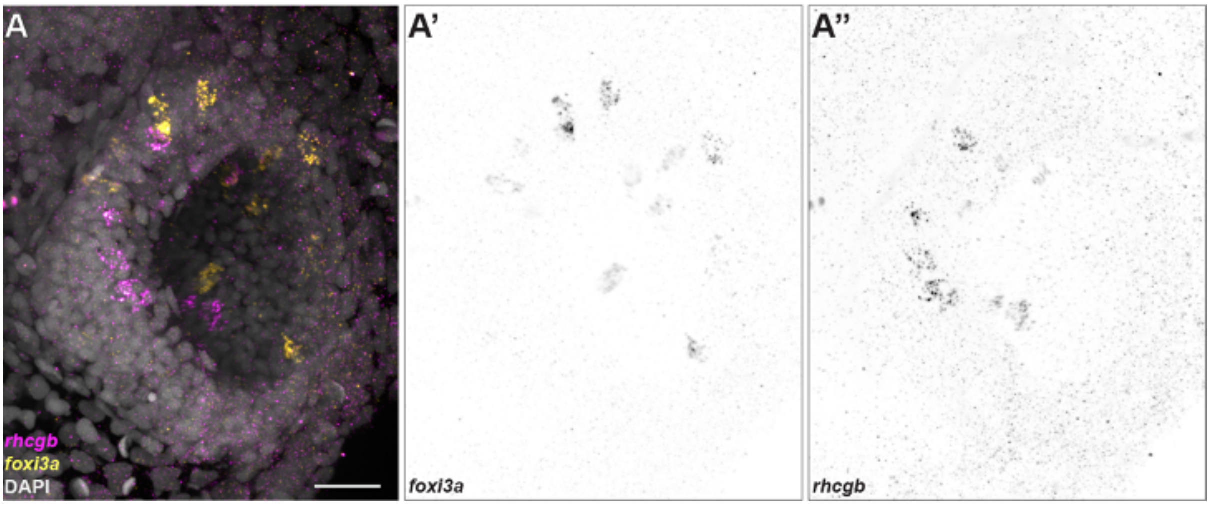

Figure S2, related to Figure 3

(A) Maximum intensity projection of a confocal image showing HCR RNA-FISH signals for *foxi3a* (yellow) and *rhcgb* (magenta) with DAPI stain (grey). **(A')** Individual channels for *foxi3a* and **(A'')** *rhcgb*. Scale bar: 20 µm.

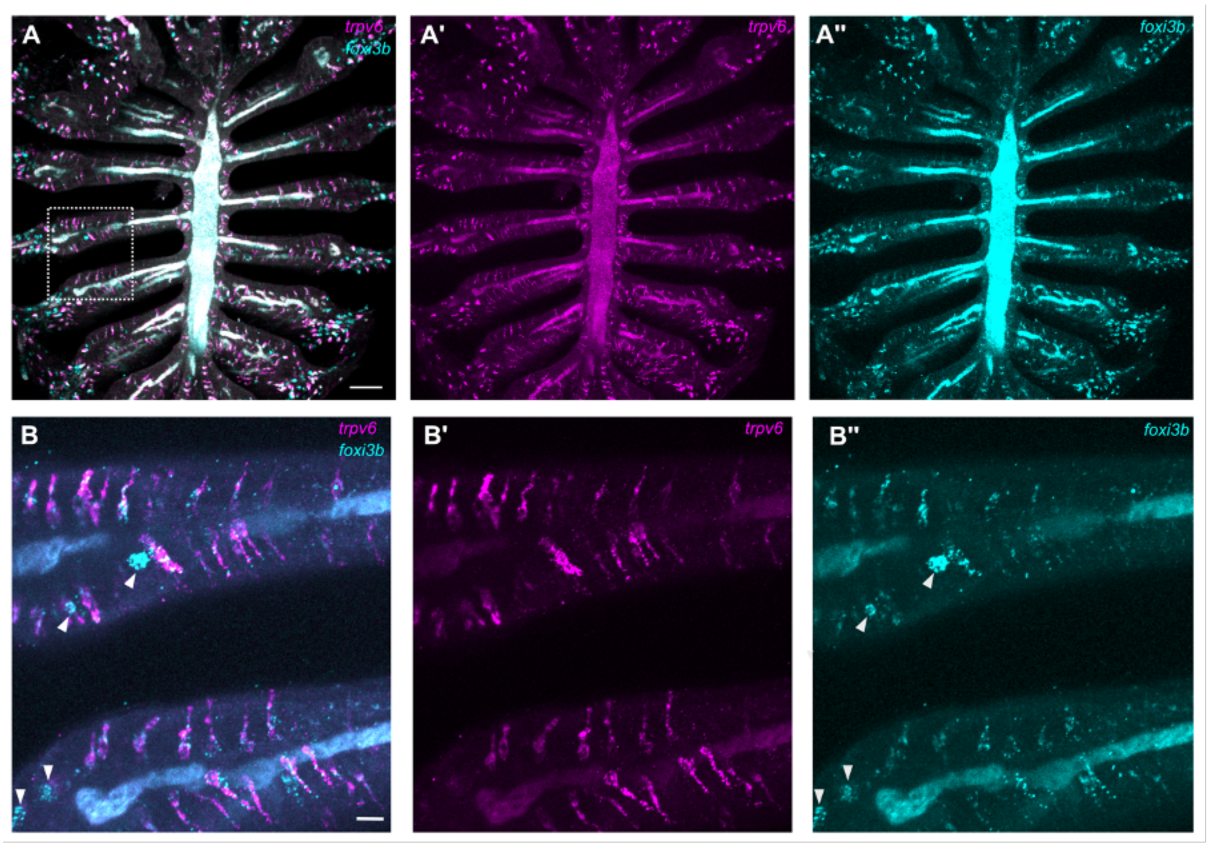

Figure S3, related to Figure 4

(A) Overview of an adult olfactory rosette, showing expression of *trpv6* **(A')** and *foxi3b* **(A'')**. **(B–B'')** High magnification view of the region outlined in panel A. The arrowheads indicate solitary NCC-like ionocytes, which express *foxi3b* and have a rounded shape. These are distinct from elongated pairs of cells that express either *foxi3b* (cyan) or *trpv6* (magenta). Scale bar: **A**, 50 µm; **B**, 20 µm.

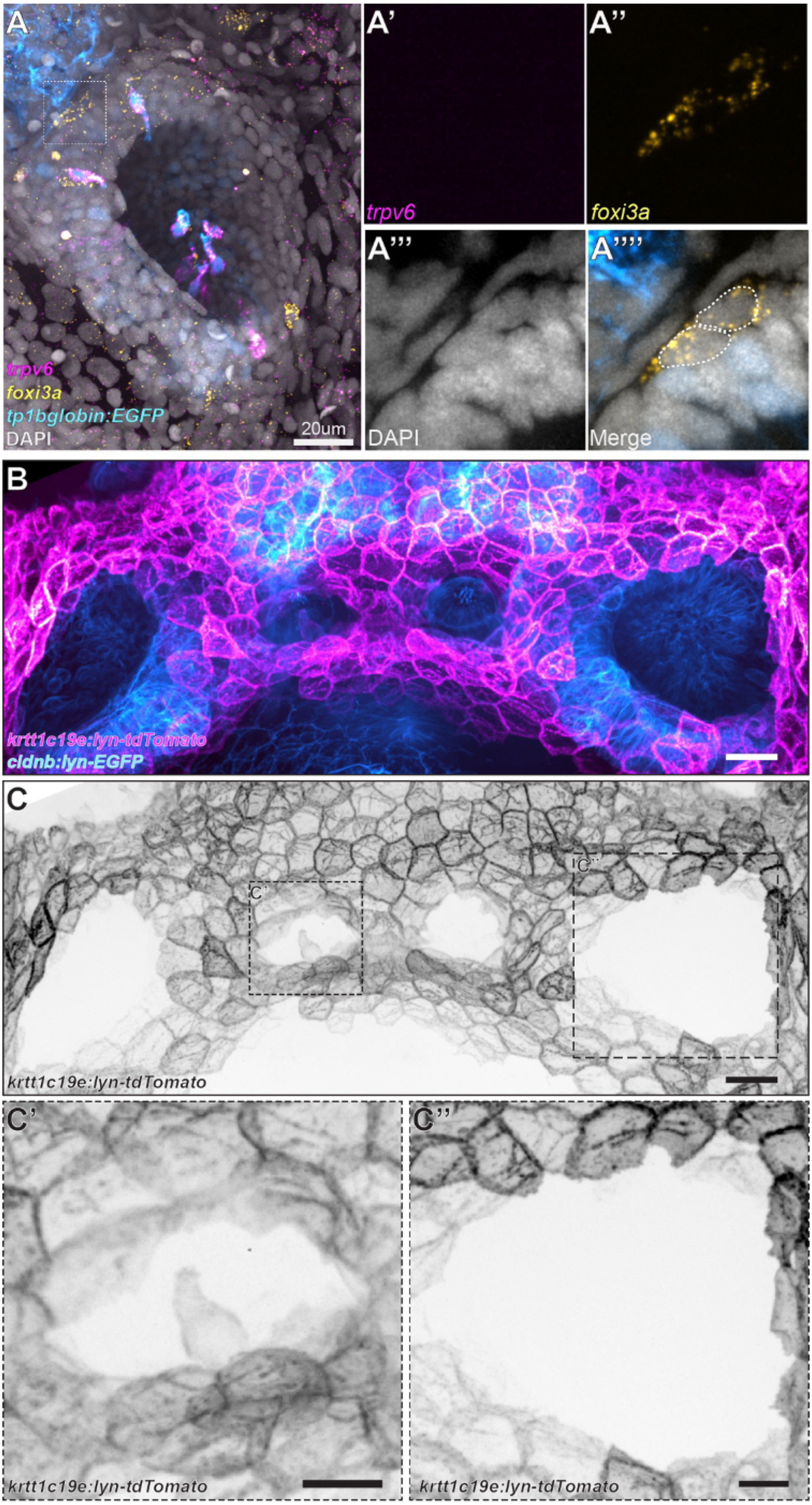
A
A'
A''
A'''
A''''
trpv6
foxi3a
tp1bglobin:EGFP
DAPI
20um
trpv6
foxi3a
DAPI
Merge
B
krtt1c19e:lyn-tdTomato
cldnb:lyn-EGFP
C
C'
C''
krtt1c19e:lyn-tdTomato
C'
C''
krtt1c19e:lyn-tdTomato
krtt1c19e:lyn-tdTomato

Figure S4, related to Figure 5

(A–A’’’) *foxi3a*$^{+}$ cells (yellow) at the edge of an olfactory pit, as shown by HCR RNA-FISH. **(B)** Maximum intensity projection of a confocal image from *Tg(-8.0cldnb:lyn-EGFP)*^{zf106Tg}*;Tg(krtt1c19e:lyn-tdTomato)*^{sq16} transgenic larva shows a pair of Nm ionocytes in the neuromast, but no tdTomato^{+} cells in the olfactory pit. **(C)** Single channel image of *krtt1c19e:lyn-tdTomato*. **(C’)** Enlargement of a neuromast containing tdTomato^{+} Nm ionocytes. **(C’’)** Enlargement of an olfactory pit containing no tdTomato^{+} cells. Scale bars: **A**, 20 μ; **B–C’’**, 5 μm.

Supplementary Table 1. Differentially expressed genes in dissected adult zebrafish olfactory organ scRNA-seq dataset

This spreadsheet contains the differentially expressed gene list (cluster markers) of olfactory cell types from the integrated adult dataset via Seurat::FindAllMarkers function with default parameters. Ionocytes are on cluster 18.

Supplementary Table 2. Differentially expressed genes in larval 5 dpf olfactory cell subset

This spreadsheet contains the differentially expressed gene list (cluster markers) of olfactory cell types from the subsetted larval dataset via Seurat::FindAllMarkers function with default parameters.

Movie 1. 3D rendering of an embryonic olfactory pit depicting HR- and NaR-like ionocytes

HCR RNA-FISH for *trpv6* (magenta) and *foxi3a* (yellow), combined with the Notch reporter *tp1bglobin:EGFP* (cyan), shows spatial distribution of ionocyte pairs in the larval 5 dpf olfactory pit. Initial image is a frontal view of the left olfactory pit, with dorsal to the top and lateral to the right. Scale bar: 15 μm.

Movie 2. 3D rendering of an embryonic olfactory pit showing NCC-like ionocytes

HCR RNA-FISH for *slc12a10.2* (yellow) and *foxi3b* (magenta), combined with the Notch reporter *tp1bglobin:EGFP* (cyan), showing the spatial distribution of ionocyte pairs in the larval 5 dpf olfactory pit. Initial image is a frontal view of the left olfactory pit, with dorsal to the top and lateral to the right. Scale bar: 15 µm.

Movie 3. New olfactory ionocytes do not express skin transgenes

Time lapse from *Tg(-8.0cldnb:lyn-EGFP)*^{zf106Tg};*Tg(krtt1c19e:lyn-tdTomato)*^{sq16} transgenic zebrafish larva showing pairs of tdTomato^{+} Nm ionocytes (arrow, middle panel) invading neuromasts, but no positive cells in the olfactory epithelium. Scale bar: 5 µm.

Movie 4. Differentiation of ionocyte pairs in a larval olfactory pit and neuromast

Time lapse from a 3 dpf transgenic zebrafish larva (*Tg(dld:hist2h2l-EGFP)*^{psi84}) showing a pair of ionocytes (cyan and yellow dots; visible at the start of the recording) invading the neuromast. At ~90 hours ionocytes are visible in the olfactory pit (red and cyan dots). No invasion was detected. Scale bar: 5 µm.

Movie 5. Ionocyte pair development in a larval olfactory pit

Time lapse from a 3 dpf transgenic zebrafish larva (*Tg(dld:hist2h2l-EGFP)*^{psi84}) showing the appearance of a pair of olfactory ionocytes (red and cyan dots). The pair is visible at approximately 85 hours, and move around together. Scale bar: 5 µm.

Movie 6. 3D reconstruction of an HR-like/NaR-like ionocyte pair from a 7 dpf wild-type olfactory pit

360° rotation of Fig. 7A. Red, HR-like cell; cyan, NaR-like cell.

Movie 7. 3D reconstruction of the tight junctions of an HR-like/NaR-like ionocyte pair

360° rotation of Fig. 7G; compare to Movie 9. Green, shallow tight junction between the two ionocytes; yellow, deep tight junction between NaR-like ionocyte and olfactory supporting cell; orange, deep tight junction between NaR-like ionocyte and multiciliated cell; blue, deep tight junction between HR-like ionocyte and olfactory supporting cell.

Movie 8. 3D reconstruction of a 3-cell ionocyte complex from a 7 dpf wild-type olfactory pit

360° rotation of Fig. 7L. Red, HR-like cell; cyan, NaR-like cell; dark blue, possible second NaR-like cell; yellow, ciliated OSN (not part of the ionocyte complex, but included for context and scale).

Movie 9. 3D reconstruction of the tight junctions between the HR-like (red) and NaR-like (cyan) ionocytes in Fig. 7L

360° rotation of Fig. 7P; compare to Movie 7. Green, shallow tight junction between the two ionocytes; yellow, deep tight junction between NaR-like ionocyte (cyan) and olfactory supporting cell; blue, deep tight junction between HR-like ionocyte (red) and olfactory supporting cell.
